# Improved participation of older people with joint contractures living in nursing homes: feasibility of study procedures in a cluster-randomised pilot trial

**DOI:** 10.1186/s13063-019-3522-1

**Published:** 2019-07-09

**Authors:** Susanne Saal, Hanna Klingshirn, Katrin Beutner, Ralf Strobl, Eva Grill, Martin Müller, Gabriele Meyer

**Affiliations:** 1 Institute of Health and Nursing Sciences, Medical Faculty, Martin Luther University, Halle-Wittenberg, Magdeburger Straße 8, 06112 Halle (Saale), Germany; 2 Institute for Medical Information Processing, Biometry and Epidemiology, Ludwig -Maximilians-University Munich, Marchioninistr 15, 81377 Munich, Germany; 3 German Center for Vertigo and Balance Disorders, Ludwig- Maximilians-University Munich, Marchioninistr. 15, 81377 Munich, Germany; 4 Faculty of Applied Health and Social Sciences, Rosenheim Technical University of Applied Sciences, Hochschulstraße 1, 83024 Rosenheim, Germany

**Keywords:** Joint contractures, Nursing homes, Participation, Complex intervention, Cluster-randomised pilot trial, Feasibility trial

## Abstract

**Background:**

Acquired joint contractures have a significant impact on functioning and quality of life in nursing home residents. There is very limited evidence on measures for prevention and treatment of disability due to joint contractures. We have developed the PECAN intervention (Participation Enabling CAre in Nursing) to improve social participation in nursing home residents. A cluster-randomised pilot trial was conducted to assess the feasibility of study procedures in preparation for a main trial according to the UK Medical Research Council (MRC) framework.

**Methods:**

Nursing homes in two regions of Germany were randomly allocated either to the intervention or optimised standard care (control group). All residents with joint contractures aged > 65 years were eligible for the study. The residents’ data were collected through structured face-to-face interviews by blinded assessors at baseline, after 3 and 6 months. The primary outcome was social participation, measured by a subscale of the PaArticular Scales. Secondary outcomes included activities and instrumental activities of daily living, health-related quality of life, falls and fall-related consequences. Data on the trial feasibility were collected via documentation forms.

**Results:**

Seven out of 12 nursing homes agreed to participate and remained in the trial. Of 265 residents who fulfilled the inclusion criteria, 129 were randomised either to the intervention (*n* = 64) or control group (*n* = 65) and analysed. A total of 109 (85%) completed the trial after 6 months. The mean age was 85.7 years (SD 7.0), 80% were women. The severity of the residents’ disability differed across the clusters. The completion rate was high (> 95%), apart from the Instrumental Activities of Daily Living Scale. Some items of the PaArticular Scales were not easily understood by residents. The frequency of falls did not differ between study groups.

**Conclusion:**

Our data confirmed the feasibility of the overall study design. We also revealed the need to improve the procedures for the recruitment of residents and for data collection before implementation into a main trial. The next step will be an adequately powered main trial to assess the effectiveness and cost-effectiveness of the intervention.

**Trial registration:**

German clinical trials register, ID: DRKS00010037. Registered on 12 February 2016.

**Electronic supplementary material:**

The online version of this article (10.1186/s13063-019-3522-1) contains supplementary material, which is available to authorized users.

## Background

Joint contractures are common among frail older people living in nursing homes [1]. Previous studies reported a prevalence of joint contractures ranging from 20 to 75% in nursing home residents [2–6]. Joint contractures are associated with restrictions in physiological joint mobility and may result in immobility [7, 8], limited capacity to perform activities of daily living (such as toileting and walking), decreased participation in social life, and increased need of nursing care [1, 3, 9, 10]. Restrictions in participation in social life are most relevant from the perspectives of both the affected individuals and the health professionals [10–12].

Despite the rising awareness of health professionals concerning joint contractures as a health problem in recent years, there is still a lack of effective measures for preventing and treating joint contractures and the associated disability [8, 13–15]. Therefore, we developed a theoretically and empirically informed complex nursing intervention, aimed at improving participation in nursing home residents with joint contractures, called the Participation Enabling CAre in Nursing intervention (PECAN) [16, 17].

In a next step, we pilot tested the PECAN intervention in a cluster-randomised controlled trial (c-RCT). We aimed to examine all of the study procedures and the feasibility of the intervention in preparation for a future definitive trial in accordance with the recommendations of the UK Medical Research Council (MRC) framework [18]. This paper presents the results of the feasibility of the study procedures in order to evaluate the design for a main trial, while the feasibility of the interventions' implementation, e.g. enablers and barriers for a successful implementation, will be reported elsewhere.

The specific objectives of this c-RCT were as follows:

1. To explore the recruitment and retention of nursing homes and residents
2. To examine the feasibility of blinding
3. To test the acceptability and eligibility of the selected outcome measures and data collection procedures
4. To assess the safety of the intervention regarding falls and fall-related fractures as unintended consequences, and
5. To explore how healthcare service utilisation data could be collected to prepare the health-economic evaluation for the main trial

## Methods

### Trial design

This multi-centre, pragmatic pilot study was designed as a two-armed, parallel-group c-RCT. A cluster was defined as one nursing home facility. A cluster design was indicated since the PECAN intervention aims to change professional behaviour in nursing staff within a specific facility.

### Participants and setting

Nursing homes were recruited in two German regions (Southeastern Bavaria and Saxony-Anhalt) from a convenience sample (existing network of cooperating practice partners). Nursing homes were invited to participate in the study via mail and a subsequent telephone call. Upon request, an onsite visit was conducted. Nursing homes were eligible if they had reported providing care for at least 25 residents with joint contractures.

Recruitment of residents started immediately after consent of the respective nursing home director. Residents were eligible if they were aged 65 years or older and with contracture of at least one joint diagnosed either by a physician, an occupational or physical therapist, or a nurse. Exclusion criteria were: terminal stage of a disease (i.e. progressive disease, poor prognosis, reduced life expectancy). For data protection purposes, the evaluation of the residents’ eligibility and the provision of written study information were carried out by the head nurse. Contact details of the resident or their legal representative (in case of the resident’s cognitive impairment) were forwarded to the researchers once the respective resident declared their interest in study participation. Finally, the resident’s or their legal representative’s written informed consent was obtained by the researchers prior to the start of the study. Although the PECAN intervention was implemented in the entire nursing home, the number of included residents was limited to 25 per cluster for feasibility reasons.

### Randomisation and blinding

Computer-generated randomisation lists were used for the allocation of clusters, stratified by region. The allocation of the clusters was performed by the external statistician, who informed the cluster representatives about the group assignment. To gather the maximum amount of information from the intervention group, more nursing homes were included in comparison to the control group [19]. All follow-up assessments were carried out by interviewers who were blinded regarding group allocation. Due to the characteristics of the intervention, it was not possible to blind nursing staff and residents. Data entry and statistical analysis was also carried out in a blinded manner.

### PECAN intervention

The focus of the PECAN intervention is to reduce barriers, to strengthen supportive environmental factors as well as to enhance personal factors, such as the residents’ motivation to maintain mobility and to engage in social activities within their current living situation.

The PECAN intervention uses a facilitation approach, which is a concerted, social process that focusses on evidence-informed practice change [20–23]. Since preliminary work revealed the absence of any robust evidence, the development of the PECAN intervention is based upon a close and iterative involvement of health professionals and residents [16].

The key aspect of the PECAN intervention to improve residents’ participation is the implementation of the biopsychosocial perspective of the International Classification of Functioning, Disability and Health (ICF) of the World Health Organization (WHO) [24] into the nursing process and the nursing home’s daily routines. This enables nurses to comprehensively assess residents’ functioning (including activities and participation) and the facilitating and hindering of contextual factors. Barriers towards participation might be modified. Actual measures depend on the local context and may contain organisational changes and changes in individual care, such as adaption of offered leisure activities or alterations in offered physical or occupational therapy, or medical aids.

**Fig. 1:**
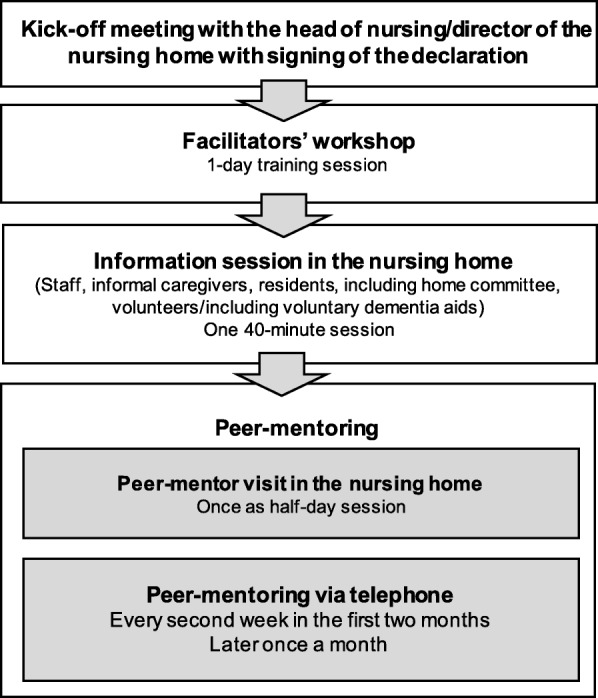
Overview of the Participation Enabling CAre in Nursing (PECAN) implementation approach

An overview of the implementation approach is displayed in Fig. 1. The implementation included the following core components:

1. *Kick-off meeting with the head nurse/nursing home director*

In a first meeting with the nursing home director and the head nurse, the policy of the intervention was introduced and discussed, and a declaration was signed to formally emphasise the institutional commitment.

2) *Facilitators’ workshop*

Facilitation is a process that depends upon the facilitator, someone who acts and enables others to implement a change in practice [20]. Nurses who were nominated as facilitators for the intervention in the nursing homes were invited to a 1-day workshop held by the researchers. During the workshop, the facilitators were trained to identify barriers against residents’ participation based on the ICF biopsychosocial model, to consider residents’ participation goals in individual care planning, to implement measures for preventing and treating joint contractures and to educate their peers with regard to the intervention.

3) *Information session*

An in-house information session lasting 40 min was held by the researchers to inform residents, family members and nursing home staff about the causes, risks and consequences of joint contractures, the PECAN intervention and its implementation approach

4) *Peer-mentoring*

The implementation approach included regular mentoring conducted by a trained nurse from the research team (the mentor) in order to support the facilitators’ role development and the planning of the implementation. At the beginning, the facilitators were visited in the nursing home by an interdisciplinary team of peer-mentors made up of the mentor, an external peer experienced in change management in nursing homes, and an occupational or physical therapist. During this visit, organisational procedures were evaluated using a checklist to identify implementation barriers and enablers. Individual care plans were critically reviewed, and changes in care were planned with support from the external peer expert.

The facilitators were supported by their mentor via telephone calls every second week throughout the first 2 months of implementation, and at least once a month thereafter.

5) *Supportive materials*

Posters and other written material informed and reminded staff, residents and their families as well as the external occupational or physical therapists and physicians. The written material comprised leaflets with information about the intervention and contact details of the facilitators and the research team. Further details of the intervention and its development are described elsewhere [16].

### Optimised standard care

In Germany, nursing homes are run by welfare organisations, communities or private operators and are financed by the German statutory long-term care insurance with additional payment from residents. According to legal requirements, 50% of nursing staff has to have 3 years of vocational training in nursing. Nursing homes usually also employ social care assistants and sometimes social workers. General practitioners, physical therapists and occupational therapists are usually not employed by the nursing home but visit the nursing homes. Technical aids are reimbursed by the German statutory long-term care insurance with additional payment by residents, whereas physiotherapy, occupational therapy and speech and language therapy are covered by the German statutory healthcare insurance with additional payment by residents. The nursing homes in the control group received an in-house information session lasting 40 min that was offered to the residents, their families and the nursing home staff. The content covered causes, risks, and consequences of joint contractures, and general information about the study.

### Data collection procedures

Interviewers were trained in structured, half-day training sessions conducted by members of the research team (HK, SuS). Data collection was carried out by structured face-to-face interviews with residents and staff. Data on the characteristics of the nursing homes were collected at baseline in an interview with the head nurse. Residents’ data were collected at baseline and at follow-up after 3 and 6 months by means of interviews and data extraction from the residents’ records.

If residents were not able to communicate (e.g. because of cognitive impairment), the interview was conducted with a proxy, i.e. a nurse in charge, using the same questionnaire items as in the residents’ interview.

#### Characteristics of nursing homes and residents

Socio-demographic and clinical data were extracted from the residents’ records. To describe the functional and cognitive status of each resident, the level of care dependency was extracted from the residents’ records. The level of care dependency is regularly assessed by expert raters from the medical service of the German statutory health insurance system using structured questionnaires and was rated as 0 = low, 1 = considerable, 2 = severe and 3 = most severe [25].

Cognitive status was determined by means of the Dementia Screening Scale (DSS) at baseline. The DSS is a valid seven-item proxy-rating tool for health professionals, comprising the two domains of memory and orientation [26]. The maximum score is 16 points (highest impairment) with a cut-off of 4 for cognitive impairment (moderate to severe dementia) [26]. In the case of cognitive impairment, a proxy version of the residents’ interview was carried out. For follow-up interviews, the DSS was repeated if the nursing staff pointed to a possible cognitive decline within the last 3 months.

#### Participation and activities (PaArticular Scales)

The PaArticular Scales, a newly developed, condition-specific and patient-centred outcome assessment based on the ICF, were assessed at baseline and after 3 and 6 months. Using two independent subscales, activity limitations (24 items, e.g. standing, grasping, dressing, eating) and participation restrictions (11 items, e.g. community life, sports, crafts, socialising) in older individuals with joint contractures can be rated as follows: none, mild or moderate, severe, or complete problems and transformed into an interval-scaled score from 0 (no problems) to 100 (complete problems) [27]. The primary outcome was measured by the participation subscale, whereas the activity subscale was a secondary outcome.

#### Instrumental Activities of Daily Living (Lawton IADL Scale)

The Lawton Instrumental Activities of Daily Living Scale (IADL Scale) is a geriatric assessment tool used to rate independent living skills in eight domains of functioning (e.g. food preparation) [28]. Each domain is represented by different items, which should resemble a resident’s highest functional level. The summary score ranges from 0 (low function) to 8 (high function). The IADL Scale was developed for older adults living independently in the community or who are in a hospital and is not recommended for use with institutionalised older adults [29]. However, in German nursing homes, in principle, there is the opportunity for residents to perform most of the instrumental activities of daily living that the IADL assesses. Hence, we included this scale to verify the activities subscale of the PaArticular Scales at baseline and after 6 months.

#### Health-related quality of life (EQ-5D-3 L)

The EQ-5D-3 L is a standardised, generic health-related quality of life questionnaire. The questionnaire consists of a descriptive three-level system based on five dimensions of health (mobility, self-care, usual activities, pain/discomfort and anxiety/depression) and includes a self-rated Visual Analogue Scale (VAS), which records self-perceived health status on a scale ranging from 0 (worst imaginable health status) to 100 (best imaginable health status) [30]. The valuation of the health status is self-rated from the resident’s point of view or is proxy-rated (version 2) by the nursing staff. Within this cluster-randomised pilot trial, the health status measured with the EQ-5D-3 L at baseline and 6-month follow-up was used to prepare the health-economic evaluation for the main trial.

#### Safety measures

Since falls might be a potential adverse event that could be attributed to the intervention, data on falls and fall-related consequences (e.g. fall-related fractures, hospital admission) were collected during the preceding 4 weeks and 6 months, at baseline and follow-up using the residents’ records.

#### Trial feasibility

Trial feasibility was evaluated using different measures. Since understanding the motivation of the nursing homes in taking part in the studies is helpful when interpreting the findings or developing tailored recruitment procedures [31], reasons for study participation (or non-participation) were evaluated by asking the head nurse. The flow of recruitment of nursing homes and residents was documented using recruitment protocols.

Retention of nursing homes and residents was documented, including reasons for early study termination. To examine whether blinding could be maintained, interviewers were asked to rate whether the visited nursing homes were allocated to the intervention or control group after each measurement point.

The acceptability and eligibility of the outcome measures were assessed by monitoring interview duration, comprehensibility of questions, and missing information (including reasons) using documentation forms after each measurement point.

#### Comparison of proxy- versus self-reported activities and participation

The level of agreement between self-reported participation and activities (PaArticular Scales) and the rating by nurses in charge was assessed at the 3-month follow-up in a sub-sample of residents without cognitive impairment. The respective interviews were conducted with residents and nurses on the same day and by the same interviewer.

#### Health-economic evaluation

Cost parameters were collected and calculated on implementation-related intervention components. Data collection procedures for outcome-related components were tested for data reliability in preparation of the health-economic evaluation in the main trial. The methodology for cost calculation followed the recommendations for the health-economic evaluations based on currently available data [32, 33]. Implementation-related resources are displayed in Additional file 1: Table S2 and were quantified using standardised protocols. Cost parameters were documented alongside the trial.

Data on utilisation of healthcare services were extracted from residents’ records or inquired about from the nursing home staff. Data were collected on the utilisation of medical and technical aids as well as on physical and occupational therapy.

### Sample size

Since this pilot c-RCT aims to assess the feasibility and acceptability rather than the effectiveness of the intervention, we did not conduct a sample size calculation [34, 35]. All analyses must, therefore, be regarded as exploratory. Based on pragmatic considerations, we planned to include a total of 150 participating residents. We assumed that an average cluster size of 25 participants is feasible, resulting in six clusters.

### Statistical analysis

Descriptive statistics were used to calculate baseline characteristics, health service utilisation, safety, and trial feasibility data. Categorical variables were summarised using absolute and relative frequencies. Continuous data were summarised using mean and standard deviation (SD). All data were stratified for the intervention and the control group. For the description of nursing homes’ characteristics, data were additionally stratified on the cluster level.

The mean differences between the intervention group and the control group starting with baseline and up to 6 months are presented along with 95% confidence intervals (CI).

The association of the primary endpoint and intervention was analysed by means of linear mixed models. The models used a mixed-effects term for varying intercepts by clusters, and for residents nested within clusters and adjusted for age and gender.

All statistical analyses were performed using R version 3.3.2 [36].

## Results

### Recruitment

Recruitment took place in February and March of 2016. Twelve nursing homes were approached, and seven agreed to participate in the study. Reasons for non-participation were lack of time (*n* = 3), no interest in the study subject (*n* = 1), and not fulfilling required self-reported joint contracture prevalence (*n* = 1). Reasons for participation (multiple reasons were possible) were professional development and further education (*n* = 5), perceiving the topic as important and interesting (*n* = 3), improving the quality of care (*n* = 3), a previous commitment to support the study (*n* = 1), collaboration with other nursing homes (*n* = 1), and anticipating legal regulations (*n* = 1).

**Fig. 2:**
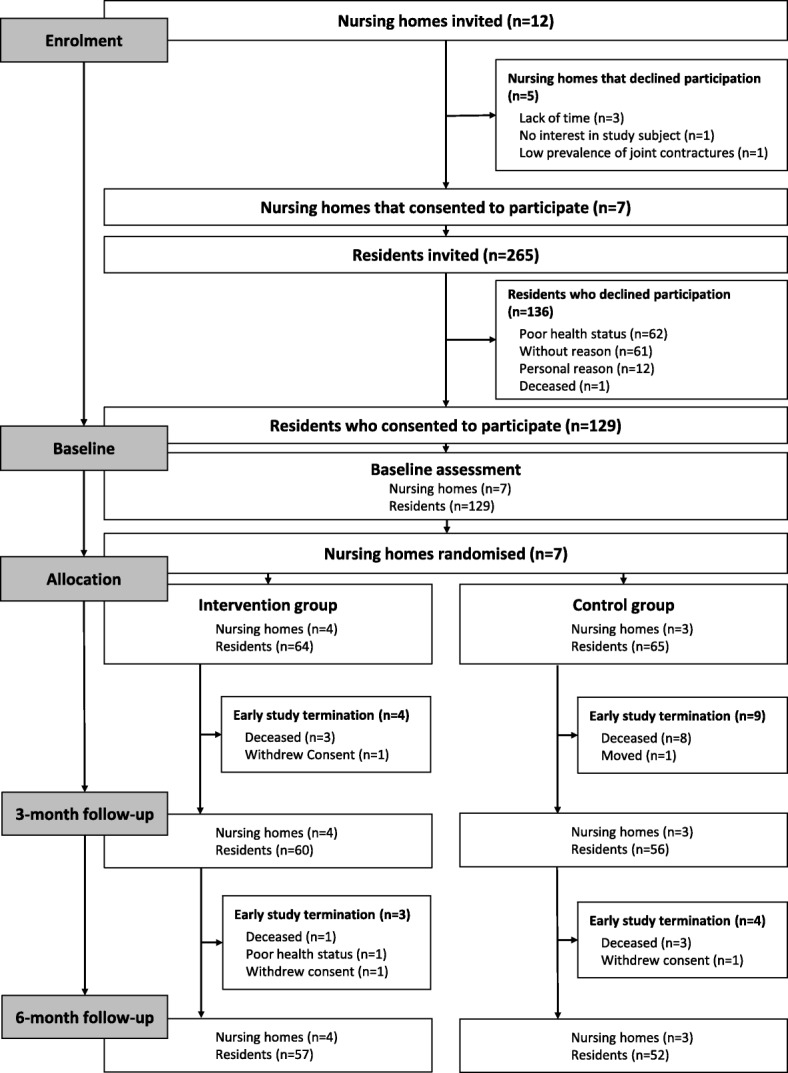
Flow of clusters and participants through the pilot trial

Among the seven participating nursing homes, a total of 265 residents met the inclusion criteria. Of these, 129 (49%) residents consented to participate. Reasons for the residents’ non-participation were poor health status (*n* = 62), personal reasons (*n* = 12), and death before inclusion (*n* = 1). A total of 61 residents gave no reason for their denial. Figure 2 displays the flow of the study.

### Baseline characteristics of nursing homes and residents

The seven nursing homes provided between 40 and 171 long-term care beds. The nursing staff to resident ratio for skilled nurses was 0.19 in total and varied from 0.16 to 0.28. The overall prevalence of joint contractures was 28% with a wide range of 19 to 96%. The nursing home characteristics are displayed in Table 1.

**Table 1:** Characteristics of nursing homes at baseline

	Intervention group				Control group			Total
	C1	C2	C3	C4	C5	C6	C7	
Study participants, *n*	9	20	11	24	24	23	18	129
Long-term care beds, *n*	40	107	171	165	48	128	115	774
Nursing home wards, *n*	3	4	4	6	2	4	6	29
Residents per nursing ward	13	27	43	28	24	32	18	27
Estimated prevalence of joint contractures	0.40	0.96	0.19	0.21	0.50	0.31	0.60	0.28
Nursing staff to resident ratio for skilled nurses and assistants	0.49	0.30	0.35	0.38	0.32	0.34	0.30	0.35
Nursing staff to resident ratio for skilled nurses	0.28	0.16	0.19	0.20	0.17	0.16	0.16	0.19

A total of 129 residents participated in the study (range: 9 to 24 per nursing home). The mean age was 85.7 years (SD = 7.0), 80% were women, and 40% were rated as severely care dependent. The level of care dependency varied between the clusters, especially for considerable (range: 4 to 70% per cluster) and most severe (range: 0 to 62% per cluster) care dependency. The mean DSS was 5.1 (SD 4.5). Half of the residents were assessed as cognitively impaired, and, therefore, 65 interviews were conducted with proxies. Cognitive status declined during the 6 months of the intervention, and a change from self-rated interview to nurse-led interview was necessary in six cases. The study groups differed in terms of the localisation of joint contractures (both extremities *n* = 36, 57% in the intervention group versus *n* = 45, 69% in the control group) and the proportion of proxy-reported assessments (*n* = 28, 44% in the intervention group versus *n* = 37, 57% in the control group). The residents’ characteristics are displayed in Table 2.

**Table 2:** Characteristics of nursing home residents at baseline

	Intervention group (*n* = 64)	Control group (*n* = 65)	Total (*n* = 129)
Age, years, mean (SD)	86.1 (6.3)	85.2 (7.7)	85.7 (7.0)
Women, *n* (%)	49 (76.6)	54 (83.1)	103 (79.8)
Localisation of joint contracture, *n* (%)			
Upper extremity	11 (17.5)	7 (10.9)	18 (14.2)
Lower extremity	16 (25.4)	13 (20.3)	29 (22.8)
Both	36 (57.1)	45 (68.8)	81 (63)
Levels of care dependency ^a^, *n* (%)			
None	1 (1.6)	0 (0)	1 (0.8)
Low	0 (0)	2 (3.1)	2 (1.6)
Considerable	23 (35.9)	18 (27.7)	41 (31.8)
Severe	24 (37.5)	27 (41.5)	51 (39.5)
Most severe	16 (25.0)	18 (27.7)	34 (26.4)
DSS, mean (SD)	4.69 (5.0)	5.46 (4.3)	5.09 (4.6)
Type of interview, *n* (%)			
Self-rated	35 (55.6)	28 (43.1)	63 (49.2)
Proxy-rated	28 (44.4)	37 (56.9)	65 (50.8)

Missing values: localisation of joint contracture (*n* = 1); Dementia Screening Scale (DSS) (*n* = 2); type of interview (*n* = 1);

^a^For the description of the functional and cognitive status, we used levels of care dependency as assessed by expert raters from the medical service of the German statutory health insurance system

### Maintenance of blinding

The study protocol could not be followed as planned as some follow-up interviews were conducted by a-priori non-blinded raters. Assessments were conducted by blinded researchers for 81 residents (70%) at the 3-month follow-up and for 74 residents (68%) at the 6-month follow-up. Three additional events of un-blinding assessors towards the cluster allocation occurred; two cases were due to unintentional disclosure of the cluster allocation by the nursing staff during the assessment visit and one case was due to unintentional disclosure of the cluster allocation by the research team. Interviewers who were asked about their perception of the grouping allocation of the clusters they visited rated the correct group allocation to 40% at the 3-month follow-up and to 70% at the 6-month follow-up.

### Retention

All seven nursing homes completed the trial. Fifteen residents died during follow-up (12%), one resident moved, one became too frail to continue (poor health status), and three withdrew their consent. Overall, 109 (84%) residents completed the trial (Fig. 2).

### Outcome measures

The effect of the PECAN intervention on participation, activities, self-perceived health status and IADL, including the number of missing values for all measurements, are presented in Table 3. The results of the participation subscale and activities subscale of the PaArticular Scales and EQ-5D-3 L indicate a slight decrease in activities, participation and self-perceived health status over 6 months, although the data imply an increase in the residents’ instrumental activities. There were no significant differences between the intervention group and the control group with regard to participation.

**Table 3:** Impact of the Participation Enabling CAre in Nursing (PECAN) intervention on participation, activity, health status, and instrumental activities of daily living

	Intervention group (*n* = 57)			Control group (*n* = 52)			Group difference^a^	LMM^b^
	Baseline Mean (SD)	6 months Mean (SD)	Difference Mean t_2_-t_0_ (SD)	Baseline Mean (SD)	6 months Mean (SD)	Difference Mean t_2_-t_0_ (SD)	Mean (95% CI)	Coefficient (95% CI)
Participation scale	46.2 (26.3)	43.0 (35.6)	− 2.9 (23.5)	43.9 (16.8)	41.3 (24.7)	− 2.4 (21.8)	0.5 (− 8.4; 9.3)	− 2.5 (− 5.5, 0.6)
Activity scale	56.5 (20.1)	54.4 (24.6)	− 2.439 (12.5)	57.5 (14.7)	51.8 (20.8)	− 5.7 (11.4)	− 3.2 (− 7.8; 1.4)	− 2.4 (− 9.8, 5.0)
VAS EQ-5D-3 L	52.9 (18.4)	51.8 (18.1)	− 2.1 (20.4)	53.9 (22.4)	54.8 (28.2)	0.7 (25.9)	2.8 (− 6.3; 11.9)	–
Lawton IADL Scale	1.5 (1.6)	2.6 (2.5)	0.6 (1.5)	1.2 (1.8)	2.2 (2.4)	0.7 (1.5)	0.1 (− 0.6; 0.7)	–

*n* = 109; t_0_ = baseline, t_2_ = 6-month follow-up

Missing values: Participation scale t_2_ (*n* = 5); Activity scale t_2_ (*n* = 3); Visual Analogue Scale of the European Quality of Life 5 Dimensions 3 Level Version

(VAS EQ-5D-3 L) t_0_ (*n* = 1), t_2_ (*n* = 4); and Lawton Instrumental Activities of Daily Living (IADL) Scale t_0_ (*n* = 18), t_2_ (*n* = 1)

Ranges: Participation scale and Activity scale 0 (no problems) to 100 (complete problems); Lawton IADL Scale 0 (low function) to 8 (high function); VAS EQ-5D-3 L 0 (worst imaginable health status) to 100 (best imaginable health status)

^a^Difference between mean-intervention (t_2_-t_0_) versus mean-control (t_2_-t_0_)

^b^Linear mixed model (LMM) with a mixed-effect term for varying intercepts by clusters, and for residents that are nested within clusters, adjusted for age and gender

### Acceptability and eligibility of the outcome measures

The interviewers’ documentation forms indicated that some items of the PaArticular Scales, especially of the subscale activities (maintaining a body position, maintaining a standing position, transferring oneself while sitting, transferring oneself while lying), were difficult for the residents to understand due to similar or overlapping contents. Additional file 1: Table S1 shows how the answers to the participation scale are distributed. The item ‘assisting people who need assistance in different areas of daily life’ was most frequently rated as ‘complete problem’, whereas the item ‘practising your religion’ was most frequently rated as ‘no problem’.

**Fig. 3:**
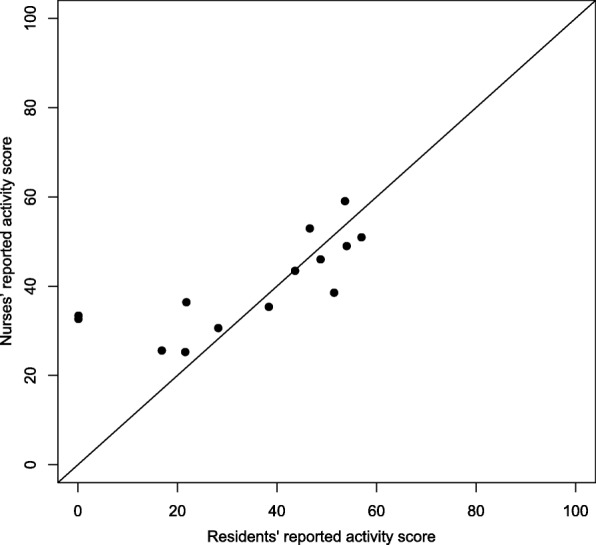
Activity scale proxy versus self-reported

**Fig. 4:**
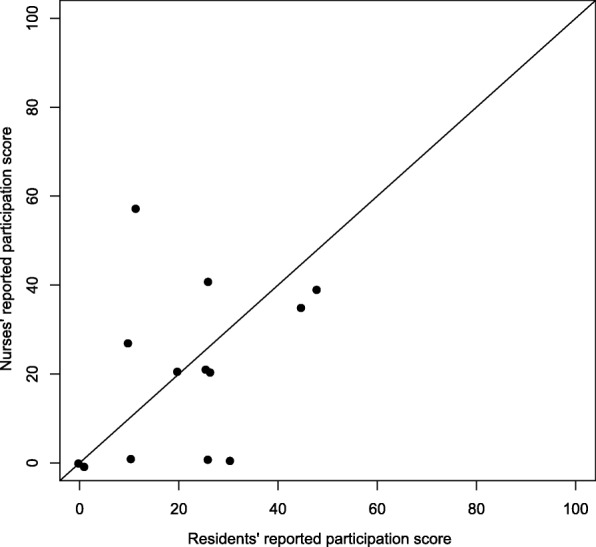
Participation scale proxy versus self-reported

At the 3-month follow-up, 14 self-reported residents’ assessments were compared to proxy assessments on the PaArticular Scales. Figures 3 and 4 provide a graphical illustration of the agreement between the ratings. Figure 3 indicates a correlation between residents’ and nurses’ rating on activities. Figure 4 fails to show any correlation between residents’ and nurses’ rating on participation.

The Lawton IADL Scale showed the highest proportion of missing values with a total number of 18 (16%). In particular, the item preparing food revealed with 15 residents (12%) the most missing values at baseline. Some residents indicated that, for example, preparing food was taken care of by the nursing home irrespective of their personal abilities, and thus, it was not relevant for them.

The EQ-5D-3 L was generally evaluated as feasible, and only a few residents needed further explanation in assessing their self-perceived health status by the VAS.

On average, the assessments took 35 min for the self-reported version and 15 min for the proxy-reported version.

### Safety

Falls and fall-related fractures during the study period are displayed in Table 4. There was no relevant difference between the intervention group and the control group concerning the frequency of falls and fall-related fractures. The number of falls remained stable throughout the follow-up.

**Table 4:** Falls and fall-related fractures during the study

	Intervention group (*n* = 57)			Control group (*n* = 52)		
	Baseline	3 months	6 months	Baseline	3 months	6 months
Residents with falls within the last 4 weeks, *n* (%)	7 (12)	7 (12)	8 (14)	2 (4)	6 (12)	5 (10)
Mean falls per resident within the last 4 weeks	1.57	1.86	1.25	1.00	1.83	1.00
Residents with falls within the last 6^a^ or 3^b^ months, *n* (%)	13^a^ (23)	12^b^ (21)	14^b^ (25)	19^a^ (37)	9^b^ (18)	11^b^ (21)
Mean falls per resident within the last 6 months	2.23	3.25	1.93	2.63	2.11	1.55
Residents with fall-related fracture, *n* (%)	2 (4)	0 (0)	0 (0)	2 (4)	1 (2)	0 (0)

*n* = 109; t_0_ = baseline, t_1_ = 3-month follow-up, t_2_ = 6-month follow-up

Missing values: mean falls per resident within the last 4 weeks t_0_ (*n* = 1); residents with falls within the last 6 t_0_ (*n* = 1) or 3 t_1_ (*n* = 1) months; and residents with fall-related fracture t_0_ (*n* = 1), t_1_ (*n* = 1)

^a^At baseline falls within the last 6 months were recorded

^b^At the 3-month follow-up and the 6-month follow-up falls within the last 3 months were recorded

### Health-economic evaluation

The total costs of the implementation-related intervention components were € 12,163.50, of which the greater part (€ 9396.20) was staff costs (Additional file 1: Table S2). The cost of the intervention per nursing home varied depending on the number and qualification of facilitators. The costs of the intervention per resident were € 109.58.

#### Utilisation of healthcare services

The following mobility aids were used by the residents at baseline: manual wheelchairs (intervention group, *n* = 23; control group, *n* = 20), electric wheelchairs (intervention group, *n* = 2; control group, *n* = 1), multi-functional wheelchairs (intervention group, *n* = 11; control group, *n* = 6), walkers (intervention group, *n* = 28; control group, *n* = 26) and walking sticks (intervention group, *n* = 3; control group, *n* = 7). At the 6-month follow-up, four manual wheelchairs (intervention group, *n* = 2; control group, *n* = 2), two multi-functional wheelchairs (intervention group, *n* = 1; control group, *n* = 1), a walker (intervention group) and a walking stick (intervention group) had been newly provided to the residents. Furthermore, two manual wheelchairs (intervention group, *n* = 1; control group, *n* = 1) and a walker (control group) could be disposed of completely.

Minutes and field notes from the interviewers indicated that medically prescribed technical and medical aids were usually not sufficiently documented in the residents’ records and information had to be obtained personally from nursing staff interviews.

Information about the provision of physical (PT) and occupational therapy (OT) was available in most of the cases (i.e. at each measurement point, less than 5% of the data were not available). In the case of PT treatment, the exact number of treatment units was available only in less than half of the cases.

### Sample size estimation for the definitive trial

Experience in the recruitment of individuals indicates that an inclusion of 15 residents per cluster is feasible. Thus, the sample size calculation was based on the assumption of a fixed cluster size of 15 residents and a free number of clusters. Using pilot data, the ICC was estimated at 0.38. This resulted in an inflation factor of (1 − (15–1) × 0.38) = 6.32. The variance observed in this pilot trial was about 200, the effect difference for the participation subscale between control and intervention group was assumed to be 10, or sometimes 12. We experienced that the PECAN intervention addressed both participation and activities and decided to use the Participation Scale and the Activities Scale as two primary endpoints simultaneously in the main trial. Since two endpoints are assessed simultaneously, a Bonferroni adjustment is performed by setting the significance level of a single test at 0.05/2 = 0.025. The size of one group in the main trial will be *n* = 241 (38 × 6.32) if the test is two-sided at a significance level of 0.025 and with a power of 80%. This results in a total of 16 clusters per study group (241/15 = 16.1). In anticipation of early study withdrawals, 15% more participants will be included, resulting in 30 clusters with a cluster size of 18 individuals and two clusters with 19 individuals; the total study size will be 578 individuals.

## Discussion

We aimed to determine the feasibility of all the study procedures in a pilot c-RCT, since it is well known that large, multi-centre, pragmatic trials are challenging, particularly in sensitive and under-explored fields of research, such as in nursing homes [37–40].

Our pilot c-RCT confirmed the feasibility of the overall study design. However, it also revealed the need to improve the procedures for the recruitment of residents and for data collection.

In contrast to other research groups who conducted trials in nursing homes [41–44], we did not experience any reluctance to participate in the study. We adopted strategies that are known to positively influence the decision-making in nursing homes with regard to participating in a study [41]. We made it clear that our intervention comes with minimum risk and possibly provides more benefits for the participants. Secondly, we emphasised the non-invasive study approach, which excluded additional costs for the nursing home staff and which we tried to keep minimally burdensome [41]. Based on recent studies involving nursing homes, we knew about the benefit of a structured, stepwise approach with timely provision of precise study information with appropriate wording for a successful enrolment [45, 46].

Some studies indicate that enrolment of nursing home residents is challenging [37–40]. Due to data protection regulations, it is not allowed to share contact data of residents with researchers without the resident’s agreement. Therefore, it was not feasible to approach eligible nursing home residents directly. Instead, the head nurses enlisted the residents. This procedure resulted in appropriate recruitment rates, since 49% of approached residents agreed to participate. However, inclusion criteria were applied differently across clusters despite the provision of a list of inclusion criteria and a personal introduction by the head nurses. In some clusters, residents with cognitive impairment were not approached. The reluctance to make decisions about research participation on behalf of residents without the capacity to consent has been known in other studies [47]. In other clusters, residents with a higher level of care dependency were predominately enrolled (cluster variation between 0% and 62% within the most severe level of care dependency).

This pilot study gave valuable information on how the enrolment procedures can be optimised. Thus, we are going to better specify the inclusion criteria for our main trial and will focus on residents with current joint contractures in major joints that are affecting their daily life and who are at least able to be mobilised into a sitting position. In accordance with the recommendations of Gismondi, additional training for the head nurse might also reduce the heterogeneous approach of the head nurses during the recruitment procedures [41]. Furthermore, in the main trial, a researcher will review the recruitment list of residents regarding the standardised application of the inclusion criteria prior to the consenting process [41]. In Table 5, we have adapted the recommendations for enrolment in nursing homes, taking our enrolment experiences into consideration [41].

**Table 5:** Adapted version of recommendations for enrolment of nursing homes according to Gismondi et al. [41]

1. Use all available state government resources, as well as professional and personal referrals, to identify and select nursing homes	
2. Long-term care institutions should be explored and recruited at the planning stage of the clinical trial so that all the necessary Institutional Review Board requirements can be met in a timely fashion	
*3. First contact with nursing home management should be initiated by the project coordinator or leading team member in charge, not by a research assistant*	
*4. Provide timely, precise study information with appropriate wording for the first nursing home contact*	
5. For more effective recruitment efforts, involve the primary care physicians (PCPs) in the nursing home as early in the process as possible. This not only helps in the identification of appropriate candidates but also encourages enrolment when the PCP agrees that the study is worthwhile	
*6. Enrolling residents should performed consecutively in one nursing ward after another instead of approaching all nursing wards simultaneously in order to keep the burden for the nursing staff as low as possible*	
7. Perform detailed patient record reviews prior to the consenting process	
8. Provide adequate training sessions and incentives to assure the cooperation of the nursing home staff	
9. Establish objective methods for the determination of mental competency as part of the protocol, and enlist the assistance of the nursing home social service staff	
10. Anticipate the need for two research team members to be present during the consenting process	
11. Reduce or eliminate any extra burden on the nursing home staff generated by the study	
13. Anticipate that state public health regulations pertaining to long-term care facilities might impede on your study procedures	
14. Collect data according to proposed, funded, and actual recruitment requirements to estimate project-specific staff time and costs	

Extended recommendations emerging from our study are shown in italics. One recommendation from Gismondi et al. 2005 about focussing on nursing homes with large bed capacities to keep the number of sites manageable was skipped since it seemed to contradict the premise to develop interventions suitable for nursing homes with both small and large bed capacities

The proportion of residents with joint contractures derived from the recruitment protocols varied vastly between the participating nursing homes, ranging from 19 to 93%. Basically, this is in line with findings from other studies where different definitions were used and hardly comparable populations were studied [2–6]. Against the background of a standardised definition of the inclusion and exclusion criteria, these findings in our pilot trial are surprising and cannot be explained by the characteristics in the nursing homes’ populations alone. We hypothesise that several components led to that phenomenon: first, a lack of awareness of joint contractures and their consequences, as well as a lack of standardised procedures for identifying joint contractures in German nursing home residents might have led to deviations from our standard procedures for inclusion and exclusion. Second, our intentionally selected broad definition of joint contractures led to the inclusion of both residents with joint contractures in small joints (e.g. joints of the fingers) and residents with joint contractures in major joints (e.g. knee or hip) and also to the inclusion of residents with multiple joint contractures (upper and lower extremities).

Blinding the interviewers was a crucial point, particularly since it was not possible to blind the participants or the staff towards the allocation [48]. Even though promotional material was handed out to nursing homes in the intervention group, it was feasible to keep the interviewers blinded. Furthermore, it proved successful to involve only one or two members of the nursing staff when arranging the interviewers’ assessment so that the risk of unmasking the group allocation is reduced. However, blinding up to the 6-month follow-up was not maintained in all clusters.

For the main effectiveness trial, we will ensure a sufficient number of interviewers to maintain the blinding, based on the experiences during the pilot c-RCT.

All seven clusters completed the trial, although the nursing homes faced several organisational problems during the study, e.g. staff turnover and staff shortages. In contrast to other studies [47], there were no differences between the intervention and control groups regarding retention. Our offer to implement PECAN after study completion might have motivated the control group to remain in the trial. Although we included both large and small nursing homes, none of the clusters reached the predefined target sample size per cluster. Therefore, sample size calculation for the main trial must take this issue into consideration.

The time used for conducting the interviews with residents and nurses seems to be acceptable. Missing data occurred in less than 5% of all assessments. This suggests appropriateness and comprehensibility of the assessment instruments with the exception of the IADL Scale (16% missing values within the baseline assessment). Although we experienced that preparing food and doing laundry were tasks that nursing home residents could generally do, only in a few cases did residents actually perform those tasks. In most cases, residents used the services offered by the nursing home. Since the items did not address the everyday life in nursing homes, we cannot recommend the IADL Scale for use in nursing home settings. The intended comparison between the subscale activities of the PaArticular Scales and the IADL was not feasible because of the high number of missing values in the IADL data. Difficulties in understanding how to complete the VAS of the EQ-5D-3 L were known from another study with nursing home residents [47] and might be improved by adding an intuitive graphical design. The PaArticular Scales were used for the first time in a c-RCT and proved to be feasible in general. Some modifications are needed since some items turned out to be less self-explanatory for the residents. More appropriate nursing-home-specific examples have to be added to the study manual.

The model of the WHO’s ICF provides clear definitions of activities and participation. “Activity is the execution of a task or action by an individual”, whereas “Participation is involvement in a life situation” [24]. In the ICF’s taxonomy, the distinction between activities and participation is less clear, in fact, it uses a common list of categories for activities and participation and provides three different solutions for the assignment of categories to either concept [49]. Considering this, together with the findings of our pilot study with only little change in both subscales, it would be reasonable to consider changes in both subscales as a positive effect of the intervention and, therefore, to define combined endpoints for the main trial.

Surprisingly, a considerable proportion of residents reported having “no problems” with most of the items of the participation subscale (Additional file 1: Table S1). This needs further explanation. According to the ICF model, activity limitations or participation restrictions have to be rated against the background of the lived experience of the individual. This means that activities or participation that are not realised in the living situation of the individual at all have to be rated as not a problem, irrespective of the objective capability. In addition, the PaArticular Scales were developed using pooled data from patients in geriatric rehabilitation facilities and nursing home residents [27]. To verify the psychometric properties of the scales in a more homogenous population, such as the trial participants in nursing homes, a further Rasch analysis using the trial data has to be carried out. This might result in a more sensitive version of the scales so that it may be possible to detect even small changes in activities and participation as a result of the developed intervention.

Another reason for only small changes in both subscales might be limitations in spreading the intervention: The intervention was delivered as planned to the facilitators, but insufficiently to the nurses, the interprofessional team and subsequently to the residents. Since this paper focusses on the feasibility of the study procedures, the findings on the feasibility of the intervention and the conclusions for improving the implementation strategy will be reported elsewhere in detail. In brief: the qualitative interviews with the facilitators, therapists, social workers, and relatives revealed a lack of involvement by the different agents regarding the overall implementation strategy. The interviewers gave possible explanations for this, mentioning, for instance, major barriers for implementing interventions, such as a lack of impact on organisational conditions and routines including unclear responsibilities, a strict separation of working areas and no established culture of contact and exchange, as well as a lack of time and staff competence.

Considering the high number of participants with cognitive impairment, instruments are needed that are appropriate for self- and proxy-reported interviews. However, differences between self- and proxy-reported outcomes are common phenomena [50–52]. Since participation is a highly individual concept, we already expected a lower agreement between the residents’ and the nurses’ rating compared to the activities scale.

Contrary to comparisons on self- and proxy-rated participation [52] and health status [50] involving next of kin, we found no tendency towards a certain direction for a lower proxy-rating. For half of the included participants, it was not feasible to involve next of kin for an interview in the nursing homes. Therefore, an assessment with the best-informed nurse is the only way to include residents with cognitive impairment in the trial. The small number of participants in our comparison (*n* = 14) allows no robust conclusion about the relation between self- and proxy-reported data. A further investigation with an adequate sample size is needed.

In terms of safety measures, i.e. the number and severity of falls, we did not document any difference between the study groups; therefore, the intervention did not seem to increase the risk of falling.

The health-economic data collection of implementation-related data generally proved to be feasible. All necessary information on prescribed technical aids and the delivery of physiotherapy and occupational therapy was not regularly documented in the residents’ records. An additional interview with nurses might be performed in the main study.

Even though our intervention consists of several components, the costs of the intervention are mainly staff costs, due to the non-productive time of the facilitators during the workshops and visits. The overall costs are lower than other similar complex intervention programs that implemented the intervention without using a facilitator [46]. However, the cost advantages of using a facilitator have to be interpreted in the context of the findings of the process evaluation, i.e. regarding the reach of the implementation approach (in preparation for publication). In addition, it should be noted that the cost findings are only preliminary. However, the health-economic evaluation approach has proved feasible and a full economic evaluation including cost utility analysis will be conducted in the main trial.

## Conclusions

Our pilot c-RCT revealed important information on how to optimise residents’ recruitment, and on blinding and data collection procedures for our planned main trial. In particular, the inclusion of nursing home residents is challenging and requires a large amount of time and detailed guidance from the study team. In the planning stage of c-RCTs in nursing homes, a tailored strategy to maintain blinding and appropriate resources of research staff are needed.

## Additional file

Additional file 1: **Table S1.** Problems in participation of residents with joint contractures during the study. **Table S2.** Resource use due to implementation of the intervention. (PDF 438 kb)

## Data Availability

The datasets analysed and the measurements used during the current study are available from the corresponding author upon request.

## Abbreviations

CI: Confidence intervals
c-RCT: Cluster-randomised controlled trial
DSS: Dementia Screening Scale
IADL: Instrumental Activities of Daily Living
ICF: International Classification of Functioning, Disability and Health of the World Health Organization
LMM: Linear mixed model
MRC: Medical Research Council
OT: Occupational therapy
PECAN: Participation Enabling CAre in Nursing
PT: Physical therapy
SD: Standard deviation
UK: United Kingdom
VAS: Visual Analogue Scale
WHO: World Health Organization
