# Supplementary material for: Improved participation of older people with joint contractures living in nursing homes: feasibility of study procedures in a cluster-randomised pilot trial
Source: Trials. 2019 Jul 9;20:411. doi: 10.1186/s13063-019-3522-1 (PMC6617884; doi:10.1186/s13063-019-3522-1)
Supplement: Supplementary file 1 — Table S1. Problems in participation of residents with joint contractures during the study. Table S2. Resource use due to implementation of the intervention. (PDF 438 kb) [file 13063_2019_3522_MOESM1_ESM.pdf]

Additional file 1

Supplement to: Saal S[1], Klingshirn H[1], Beutner K, Strobl R, Grill E, Müller M[2], Meyer G[2]. Improved participation of older people with joint contractures living in nursing homes: Feasibility of study procedures in a cluster-randomised pilot trial.

[1] shared first authorship

[2] shared senior authorship

Table A1 Problems in participation of residents with joint contractures during the study

Code [a] / Item			Intervention group (n=57)		Control group (n=52)	
			Baseline	6 months	Baseline	6 months
d660	Assisting others	No	20 (35)	23 (40)	14 (27)	17 (33%)
		Mild or moderate	0 (0)	5 (9)	4 (8)	3 (6)
		Severe	5 (9)	6 (11)	5 (10)	3 (6)
		Complete	32 (56)	23 (40)	29 (56)	28 (55)
d7105	Physical contact in relationships	No	24 (42)	33 (58)	18 (35)	38 (73)
		Mild or moderate	11 (19)	4 (7)	18 (35)	1 (2)
		Severe	11 (19)	6 (11)	11 (21)	5 (10)
		Complete	11 (19)	14 (25)	5 (10)	8 (15)
d750	Informal social relationships	No	26 (46)	31 (54)	17 (33)	28 (54)
		Mild or moderate	9 (16)	4 (7)	20 (38)	3 (6)
		Severe	7 (12)	3 (5)	6 (12)	5 (10)
		Complete	15 (26)	19 (33)	9 (17)	16 (31)
d910	Community life	No	22 (39)	26 (46)	22 (43)	19 (37)
		Mild or moderate	8 (14)	4 (7)	7 (14)	4 (8)
		Severe	16 (28)	5 (9)	13 (25)	2 (4)
		Complete	11 (19)	21 (38)	9 (18)	26 (51)
d9200	Playing	No	22 (39)	35 (61)	25 (48)	26 (50)
		Mild or moderate	11 (19)	1 (2)	8 (15)	3 (6)
		Severe	10 (18)	3 (5)	8 (15)	3 (6)
		Complete	14 (25)	18 (32)	11 (21)	20 (38)
d9201	Sports	No	17 (30)	26 (46)	15 (29)	23 (45)
		Mild or moderate	11 (19)	4 (7)	10 (19)	7 (14)
		Severe	15 (26)	10 (18)	9 (17)	4 (8)
		Complete	14 (25)	17 (30)	18 (35)	17 (33)
d9202	Arts and culture	No	20 (35)	28 (49)	22 (42)	28 (54)
		Mild or moderate	5 (9)	5 (9)	10 (19)	4 (8)
		Severe	12 (21)	4 (7)	12 (23)	7 (13)
		Complete	20 (35)	20 (35)	8 (15)	13 (25)
d9203	Crafts	No	16 (28)	24 (42)	17 (33)	19 (37)
		Mild or moderate	2 (4)	3 (5)	7 (13)	0 (0)
		Severe	12 (21)	7 (12)	6 (12)	8 (15)
		Complete	27 (47)	23 (40)	22 (42)	25 (48)
d9204	Hobbies	No	15 (26)	26 (46)	17 (33)	27 (53)
		Mild or moderate	5 (9)	4 (7)	5 (10)	0 (0)
		Severe	10 (18)	5 (9)	12 (24)	6 (12)

		Complete	27 (47)	22 (39)	17 (33)	18 (35)
d9205	Socializing	No	19 (33)	34 (60)	17 (33)	31 (60)
		Mild or moderate	10 (18)	4 (7)	12 (23)	4 (8)
		Severe	21 (37)	3 (5)	16 (31)	3 (6)
		Complete	7 (12)	16 (28)	7 (13)	14 (27)
d930	Religion and spirituality	No	32 (56)	36 (63)	47 (90)	47 (90)
		Mild or moderate	7 (12)	3 (5)	3 (6)	1 (2)
		Severe	9 (16)	4 (7)	2 (4)	1 (2)
		Complete	9 (16)	14 (25)	0 (0)	3 (6)

[a] Code in the first column corresponding to the respective category of the International Classification of Functioning, Disability and Health. Values are n (%)

Table 2 -Resource use due to implementation of the intervention

**Item of resource**	**Unit of measure**	**Unit cost (€)**	**Total cost (€)**	**Mean cost per intervention cluster (€)**	**Source**
Advertising material/brief information	Item	6.93	865.36	123.63	Invoice by the manufacturer
Education material	Item	9.75	351.12	50.16	Invoice by the manufacturer
Room rental	Hour	12.50	553.44	79.06	Finance department of the Martin Luther University Halle-Wittenberg and the Ludwig-Maximilians-University Munich
Workshop catering	Participant	20.03	388.08	55.44	Invoice by the manufacturer
Travel expenses	Kilometer lump sum	0.30	609.30	87.04	Finance department of the Martin Luther University Halle-Wittenberg
External consultants‘ fee	Lump sum	3700.00	3700.00	528.57	Invoice by the Martin Luther University Halle-Wittenberg
Peer mentors‘ salary	Minute	0.32	150.40	21.49	Finance department of the Martin Luther University Halle-Wittenberg and the Ludwig-Maximilians-University Munich
Investigators‘ salary (total)	Hour	24.87	1840.64	262.95	Finance department of the Martin Luther University Halle-Wittenberg and the Ludwig-Maximilians-University Munich
Facilitators‘ salary (total)	Hour	23.48	3705.16	529.31	Finance department of nursing homes
**Sum**			**12163.50**	**1756.85**	

**Per resident (n=111)**		**109.58**		
